# Horizontal ridge augmentation in maxillary esthetic zone using bovine bone graft versus extended sticky bone with simultaneous implant placement: a randomized clinical study

**DOI:** 10.1186/s12903-026-09431-y

**Published:** 2026-09-09

**Authors:** Amira A. M.M. Attia, Mohammed A. Abass, Mohammed Talaat Salem, Wael M. Said Ahmed

**Affiliations:** 1 Oral and Maxillofacial surgery Department, Faculty of Dentistry, Mansoura University, Mansoura, Egypt; 2 Faculty of Dentistry, Mansoura University, Mansoura, Egypt

**Keywords:** Medpark, Extended sticky bone, Horizontal ridge augmentation, Soft tissue healing, Implant stability, Bone volume

## Abstract

**Background:**

Horizontal ridge deficiency in the anterior maxilla may compromise optimal implant placement and esthetic outcomes. This randomized clinical trial evaluated the clinical and radiographic outcomes of bovine bone graft (Medpark)^®^ compared with extended sticky bone (ESB) for horizontal ridge augmentation with simultaneous implant placement.

**Materials and methods:**

Sixteen patients with missed maxillary anterior tooth and horizontal ridge resorption were randomly allocated into two groups (*n* = 8 each). Group I received Medpark graft, while Group II received ESB with simultaneous implant placement. Implant stability was assessed immediately and after 6 months. Soft tissue healing was evaluated using the Landry healing index at 2 and 4 weeks. Cone-beam computed tomography (CBCT) was performed preoperatively (T0), immediately postoperatively (T1), and at 6 months (T6) to assess volumetric bone changes. Data were statistically analyzed using SPSS software (*p* ≤ 0.05).

**Results:**

All implants survived without complications. Implant stability increased significantly in both groups at 6 months (*p* = 0.001), with no significant intergroup differences. Soft tissue healing improved significantly over time in both groups (*p* = 0.008), without significant differences between them. Both groups demonstrated significant bone volume gain at T1 followed by partial reduction at T6, remaining above baseline. A significant difference was observed at T1 in favor of ESB (*p* = 0.001), while greater bone loss from T1 to T6 was noted in the ESB group.

**Conclusion:**

Within the limitations of this study, both Medpark and extended sticky bone demonstrated favorable short-term outcomes for horizontal ridge augmentation. Although extended sticky bone achieved greater immediate postoperative bone volume gain, greater subsequent resorption resulted in comparable clinical and radiographic outcomes between the two groups after 6 months. Further studies with larger sample sizes and longer follow-up periods are required to determine the long-term clinical significance of these findings.

**Trial registration:**

The study was listed on www.clinicaltrials.gov with registration number (NCT07589426) on 03/05/2026.

## Background

Tooth extraction is commonly followed by physiological bone remodeling that results in horizontal and vertical ridge reduction, particularly in the anterior maxilla [1–3]. This resorption is more pronounced on the buccal aspect, often leading to horizontal ridge deficiencies that may compromise ideal implant positioning and esthetic outcomes, particularly in the maxillary esthetic zone [2]. 

To overcome these limitations, various ridge augmentation techniques have been developed, including guided bone regeneration, onlay grafting, titanium mesh, and minimally invasive augmentation approaches [4–6]. 

Among grafting materials, xenogeneic bone substitutes have been widely used as alternatives to autogenous grafts due to their osteoconductive properties and biocompatibility [7]. Medpark is a xenogeneic bone graft material composed primarily of deproteinized bovine cancellous bone and hydroxyapatite. In addition, it contains a hydrogel agent that becomes viscous upon hydration, allowing the graft to acquire a moldable sticky-bone consistency. This self-hardening characteristic enhances graft stability, handling, and space-maintaining ability during regenerative procedures. The material can be easily shaped and adapted to various defect morphologies, allowing clinicians to achieve a customized augmentation according to individual anatomical requirements. Medpark is osteoconductive and cytocompatible, acting as a scaffold that facilitates the formation of new bone by host-derived osteogenic cells. Also, the porous microarchitecture of Medpark (> 70%) allows rapid hydration and enhances its regenerative performance by increasing surface area and allowing better cell attachment and vascular ingrowth. Its interconnected pores support osteoconduction by facilitating the migration of osteoblasts and mesenchymal stem cells within the graft. In addition, the rough surface improves cellular adhesion and promotes early bone formation [8]. According to histological analyses, xenogenic grafts gradually resorb and are replaced by freshly grown bone tissue, returning the surrounding bone and tissue to their original esthetic forms [9–11]. 

In parallel, platelet concentrates have gained increasing attention in regenerative dentistry due to their capacity to enhance healing through the sustained release of growth factors and promotion of angiogenesis [12, 13]. 

Sticky Bone, a growth factor-rich grafting matrix, is a bone graft material embedded within a fibrin matrix. It is firm, moldable, adaptable to various defect morphologies, and can resist micromotion, which is critical for maintaining graft volume. Furthermore, it serves as a barrier that prevents soft tissue from growing into the grafted site [14]. 

However, this firmness typically decreases within 8 to 12 days as the fibrin matrix begins to resorb. To overcome this shortage, an enhanced version known as “Extended Sticky Bone” was developed under the name Bio-Bone. This biomaterial integrates Alb-gel, particulate bone graft, and injectable Platelet-Rich Fibrin (PRF) to form a matrix that sustains biological activity up to 4 to 6 months. Owing to this extended viability, Bio-Bone can be utilized in augmentative bone procedures without the need for an additional collagen membrane, thereby reducing the overall cost of treatment [15]. 

Despite the growing use of both xenogeneic grafts and platelet-based composites, limited clinical evidence is available comparing their outcomes when used for horizontal ridge augmentation with simultaneous implant placement in the anterior maxilla [16]. 

The primary outcome of this randomized clinical trial was the change in augmented bone volume assessed radiographically using CBCT. Secondary outcomes included implant stability, soft tissue healing, and implant survival. We hypothesized that extended sticky bone would achieve greater immediate postoperative volumetric augmentation than Medpark bovine bone graft while maintaining comparable short-term clinical outcomes following simultaneous implant placement in the anterior maxilla.

## Patients and methods

### Ethical statement

This study was designed as a parallel-arm randomized clinical trial conducted at the Oral and Maxillofacial Surgery Department, Faculty of Dentistry, Mansoura University, Egypt. The study protocol received ethical approval from the Institutional Review Board of the Faculty of Dentistry, Mansoura University (Approval No. Ms.25.04.21). The study adhered to the principles of the Declaration of Helsinki and was reported in accordance with CONSORT guidelines [17]. Written informed consent was obtained from all participants prior to enrollment. The trial was registered at ClinicalTrials.gov with registration number (NCT07589426) on 03/05/2026.

### Patient selection

A total of 16 patients requiring implant rehabilitation of missing maxillary anterior teeth with associated horizontal ridge deficiency were recruited from the outpatient clinic of the Oral and Maxillofacial Surgery Department, Faculty of Dentistry, Mansoura University, Egypt.

### Inclusion criteria

Patients aged 18–45 years, good oral hygiene, presence of horizontal ridge deficiency in the anterior maxilla (Siebert Class I) [18], No deep undercut at the ridge in the buccal aspect, ≥ 3 mm keratinized gingiva [19]., adequate inter-arch space [20], and the ability to comply with follow-up visits.

### Exclusion criteria

Patients with systemic conditions contraindicating surgery or implant placement, smokers, pregnant women, parafunctional habits (e.g., bruxism), or local pathological conditions affecting bone healing.

### Sample size calculation

The sample size was calculated by the G-power program (3.1.9.4) based on previous studies [21, 22], using a two-tailed t-test ( Means: Wilcoxon-Mann-Whitney test (two groups), using an effect size of 1.79, α = 0.05, and 80% power, yielding a total sample size of 14 (7 per group). We added extra one subject to compensate for the dropout.

### Randomization method

For this trial with Medpark and extended sticky bone groups involving 16 subjects (8 per group), Participants were randomly allocated to either the Medpark or extended sticky bone group using a computer-generated block randomization sequence with a fixed block size of four. The randomization list was prepared by an independent investigator who was not involved in patient enrollment, surgical treatment, or outcome assessment.

### Allocation concealment

To ensure allocation concealment, the randomization sequence was hidden from the surgical team using sequentially numbered, opaque, sealed envelopes. A designated clinical assistant, who was not involved in the surgical procedures or outcome assessments, opened the corresponding envelope to reveal the group assignment only after the flap elevation and confirmation of the suitability of the site for implant placement to reveal the group assignment.

### Blinding

Because of the obvious differences between the grafting materials, blinding of the operating surgeon was not feasible. However, all outcome assessments were performed by investigators blinded to treatment allocation. CBCT datasets were anonymized before volumetric analysis, Osstell measurements were obtained by a blinded operator, and soft tissue healing was evaluated using the Landry index by an independent blinded assessor.

### Patients’ grouping

The sixteen patients were randomly divided into two equal groups:

- *In Group I*: Eight dental implants were inserted simultaneously with horizontal ridge augmentation using Medpark bone graft.
- *In Group II*: Eight dental implants were inserted simultaneously with horizontal ridge augmentation using ESB.

### Surgical protocol

All surgical procedures were performed under strict aseptic conditions by the same experienced surgeon.

### Preoperative preparation

**Fig. 1:**
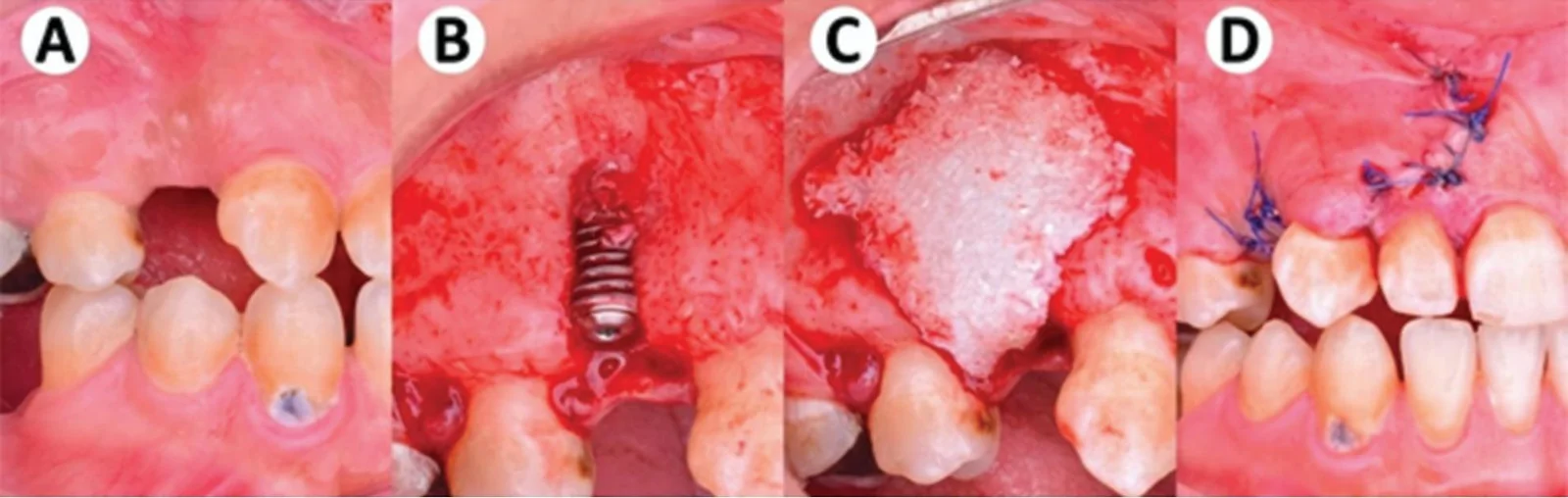
group I; (**A**), Preoperative frontal view (**B**), alveolar ridge defect after placement of dental implant (**C**), Horizontal ridge augmentation with Medpark bone graft (**D**), Flap reapproximation and suturing

**Fig. 2:**
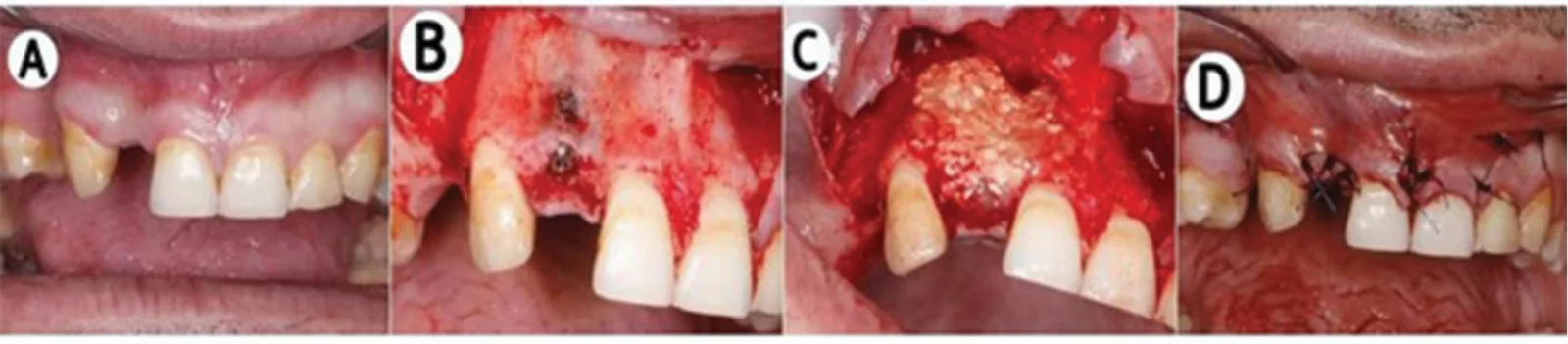
Group II; (**A**), Preoperative frontal view (**B**), alveolar ridge defect after placement of dental implant (**C**), Horizontal ridge augmentation with Extended sticky bone graft (**D**), Flap reapproximation and suturing

Preoperative assessment included medical and dental history, clinical examination, study casts, and radiographic evaluation using panoramic radiography and CBCT for treatment planning. Baseline intraoral photographs were taken. (Fig. 1A & Fig. 2A). Patients received prophylactic antibiotic therapy amoxicillin–clavulanic acid (Augmentin 1 gm tablets, GlaxoSmithKline Pharmaceuticals, Egypt) 1 h before surgery. Preoperative oral rinse with 0.12% chlorhexidine (Listermix Plus mouthwash, SIGMA, Egypt) was performed for 1 min.

### Surgical procedure

Local anesthesia was administered using articaine hydrochloride 4% with epinephrine (1:100,000). A mid-crestal incision with vertical releasing incisions was performed, followed by elevation of a full-thickness mucoperiosteal flap to expose the alveolar ridge.

Osteotomy preparation was carried out according to the manufacturer’s protocol under copious saline irrigation, and dental implants (NeoBiotech Implant Neobiotech Co.) were placed in the planned prosthetic position **(**Figs. 1B and 2B**)**. Primary implant stability was assessed immediately after placement, and cover screws were subsequently placed.

Following implant placement, horizontal ridge augmentation was performed as follows:

- *For group I*: Medpark bone graft (Medpark Regeneration Yeongdeungpo-gu, Seoul, Republic of Korea) was hydrated and adapted to the defect site to augment ridge width (Fig. 1C)
- *For group II*: PRF and ALB-PRF were prepared by centrifuging 9 ml of blood at 2700 rpm for 8 minutes. The platelet-poor plasma was thermally denatured and mixed with liquid PRF and xenograft( GARANTIE, GmbH, Germany) (equal amount of medpark) to create ESB, which was applied around the implant to reconstruct the horizontal ridge defect [23].

In both groups, graft material was carefully contoured, and primary wound closure was achieved using interrupted and mattress sutures without tension (Figs. 1D and 2D).

### Postoperative care

Postoperative instructions included cold application during the first 24 h and avoidance of mechanical trauma to the surgical site. Antibiotics were prescribed for 5 days, and analgesics (diclofenac potassium 50 mg) were given as needed. Patients were instructed to use 0.12% chlorhexidine mouthwash twice daily for 7 days.

Sutures were removed after 10–14 days. A second-stage surgery was performed after 6 months to expose the implants and place healing abutments. After two weeks, the healing abutments were removed, and a digital impression was obtained to form cement retained zirconia crowns.

### Evaluations

**Fig. 3:**
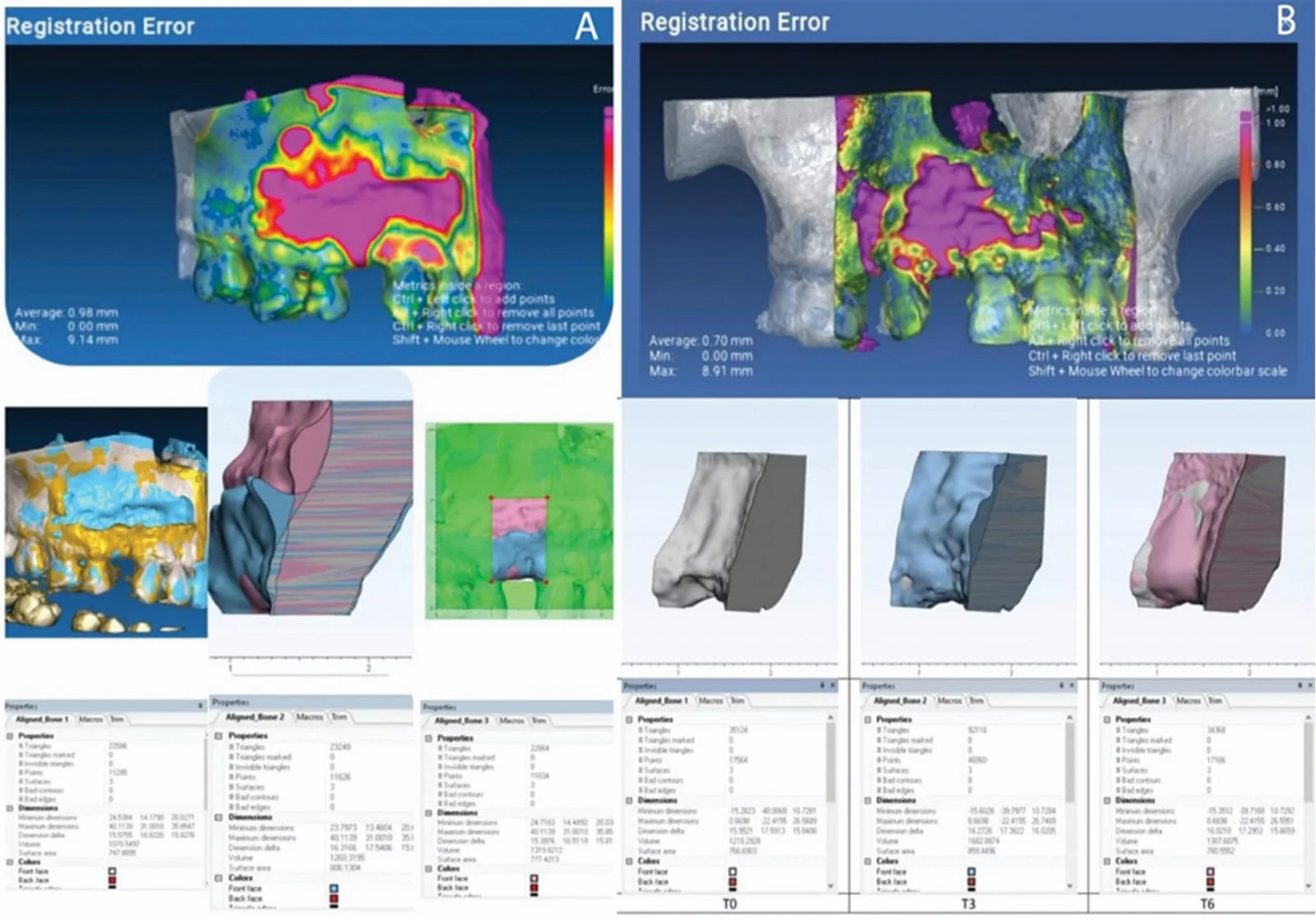
(**A** &** B**): volumetric analysis at three assessment time points

- *Clinical evaluations*:
  1. *Implant stability*: Implant stability was assessed immediately after implant placement and at 6 months using RFA with an Osstell device. Measurements were expressed as Implant Stability Quotient (ISQ) values. Stability was categorized as high (ISQ >70), moderate (ISQ 60–69), or low (ISQ <60) [24–26].
  2. *Soft tissue evaluation*: Soft tissue healing at the surgical site was assessed using the Landry healing index [27] at 2 and 4 weeks postoperatively. This index evaluates wound healing based on five clinical parameters: tissue color, bleeding on palpation, presence of granulation tissue, condition of the incision margin, and degree of epithelialization. Healing was scored on a 5-point scale as follows: 
    *Score 1 (very poor)*: ≥2 adverse findings such as redness or cyanosis, absence of granulation tissue, bleeding on palpation, suppuration, or lack of epithelialization
    *Score 2 (poor)*: presence of one adverse finding (e.g., redness, suppuration, or incomplete epithelialization)
    *Score 3 (fair)*: slightly red tissue, partial epithelialization, presence of granulation tissue, and no bleeding or suppuration
    *Score 4 (good)*: pink tissue, nearly complete epithelialization, healthy granulation tissue, and absence of bleeding or suppuration
    *Score 5 (excellent)*: pink tissue with complete epithelialization, no bleeding on palpation, and no suppuration

b. Radiographic evaluation:

Volumetric bone analysis [28]:

- Radiographic evaluation of bone volume was performed using CBCT at three time points: preoperatively (T0), immediately postoperatively within one week (T1), and at 6 months (T6).
- CBCT datasets were exported in DICOM format and processed using dedicated 3D analysis software (RealGuide version 5.3, 3Diemme, Italy). Segmentation of the maxillary bone was performed using a standardized gray value threshold, followed by manual refinement and filling of internal voids. The segmented models were then converted into STL files.
- The STL models from T0, T1, and T6 were superimposed using a surface-based registration method to ensure accurate spatial alignment. Bone volume was measured in mm3, CBCT segmentation threshold for bone was 500-3000 HU, and the registration error was 0 to 0.8 mm. Standardized cross-sectional slices were obtained at the implant sites to allow reproducible measurements (Fig. 3A, Fig. 3B).

The following volumetric parameters were calculated:

- *Bone volume at each time point (T0, T1, T6)*.
- *Bone volume gain*: difference between T1 and T0, and between T6 and T0.
- *Bone volume loss*: difference between T6 and T1.

### Statistical analysis

Data were analyzed using SPSS software (version 26.0; IBM Corp., Armonk, NY, USA). Data normality was assessed using the Shapiro–Wilk test. Continuous variables with a normal distribution were expressed as mean ± standard deviation and analyzed using independent-samples and paired-samples t-tests, whereas non-normally distributed data were analyzed using the Mann–Whitney U test and Wilcoxon signed-rank test, as appropriate. Categorical variables were analyzed using the Chi-square or Fisher’s exact test. The primary outcome was postoperative volumetric bone gain assessed by CBCT, while implant stability, soft tissue healing, and implant survival were considered secondary outcomes. Statistical significance was established at *p* ≤ 0.05.

## Results

A total of 17 patients were initially assessed for eligibility, of whom 1 was excluded for not meeting the inclusion criteria. The remaining 16 patients were randomly allocated into two equal groups (*n* = 8 each).

**Fig. 4:**
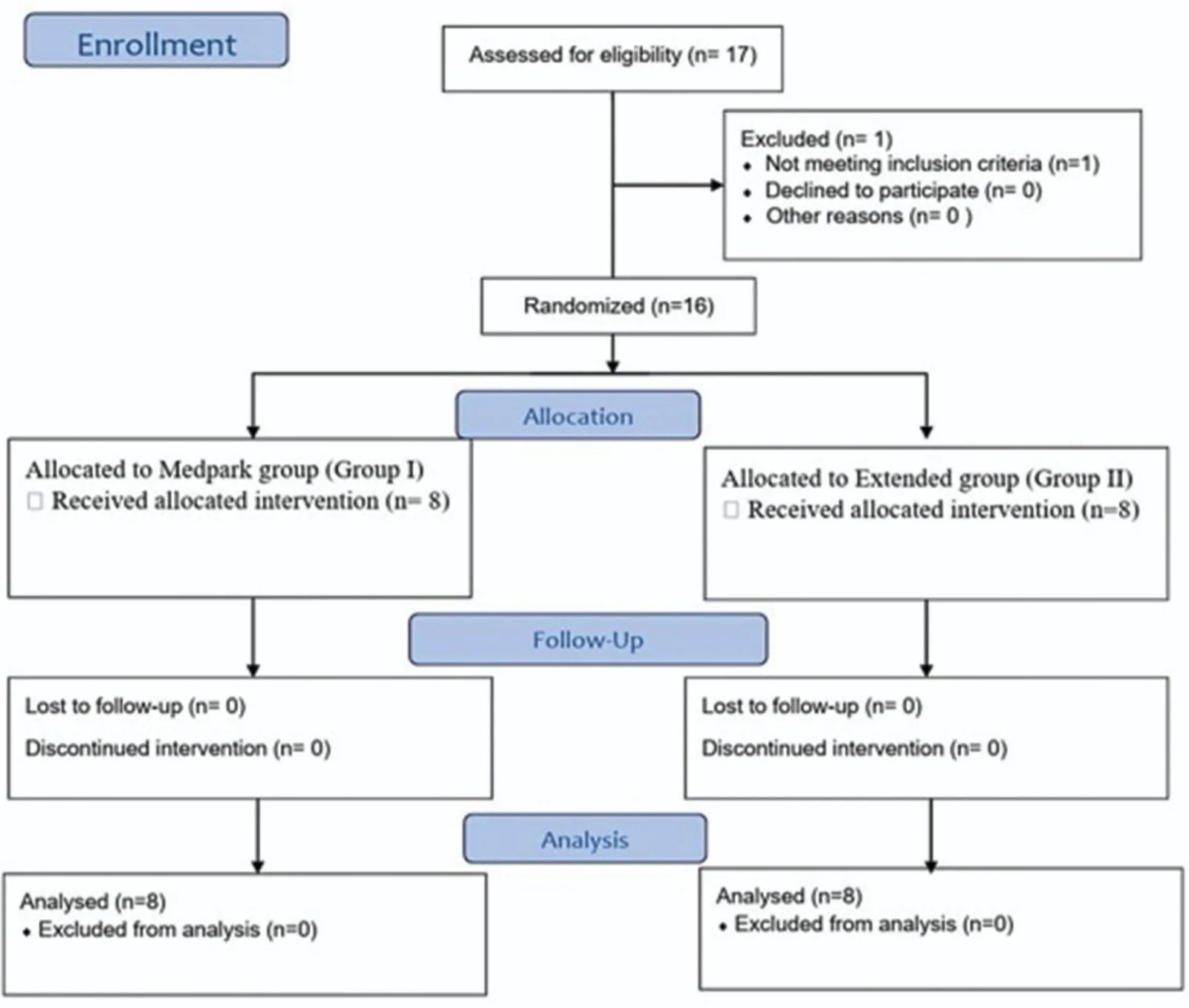
Flow Chart according to the CONSORT guidelines

All patients completed the 6-month follow-up period. All patients received the assigned intervention, with no dropouts or losses to follow-up, resulting in a 100% completion rate. No intraoperative or postoperative complications were reported, and all implants achieved successful osseointegration (100% survival rate). The flow of participants throughout the study is illustrated in the CONSORT flow diagram (Fig. 4).

### Demographic data and implant size

As shown in Table 1, the mean age was 30.71 ± 6.99 years in Group I and 29.28 ± 3.99 years in group II, with no statistically significant difference between groups (*p* = 0.647). Regarding gender distribution, females represented 87.5%in group I and 50.0% in group II, with no statistically significant difference (*p* = 0.282).

All implants were standardized in terms of dimensions (10 mm length and 4 mm diameter).

**Table 1:** Demographic characters and implant size among studied groups

	Group I *N* = 8	Group II *N* = 8	Test of significance
Age (years)	30.71 ± 6.99	29.28 ± 3.99	t = 0.469
Mean ± SD			*P* = 0.647
Sex n (%)			
Females	7 (87.5%)	4 (50.0%)	ꭓ^2^= 0.282
Males	1 (12.5%)	4 (50.0%)	*P* = 0.282
Implant length (mm)	10 ± 0.0	10 ± 0.0	
implant width (mm)	4 ± 0.0	4 ± 0.0	

*SD* standard deviation Between-group comparisons for continuous variables (Age) used independent t-tests

*t* Student t test, ꭓ^2^Chi-Square test

### Clinical outcomes

**Fig. 5:**
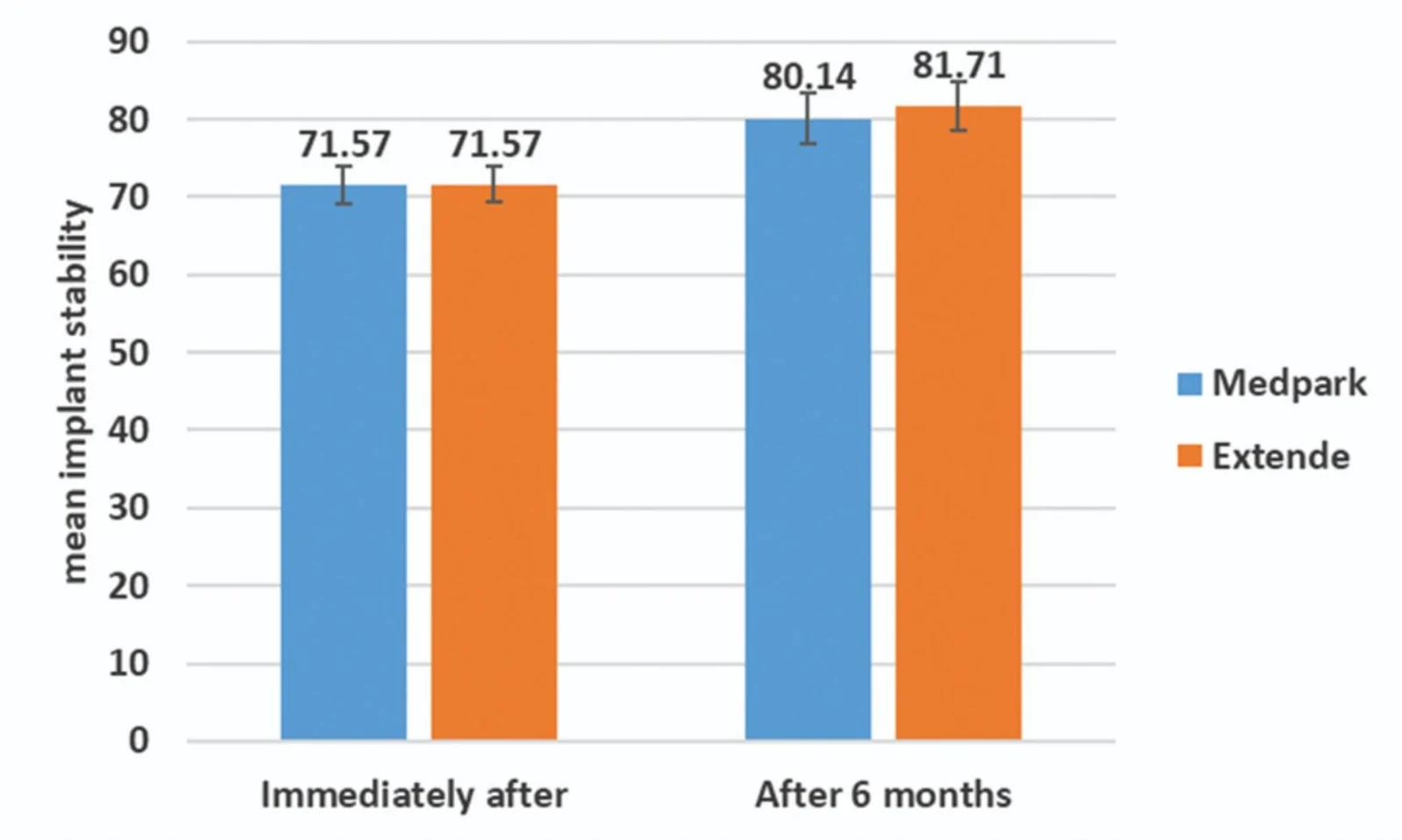
Bar chart showing comparison of implant stability between studied groups and during follow-up

1. Implant stability
  *All implants survived throughout the follow-up period, yielding a 100% survival rate.
  *The mean implant stability immediately after placement was 71.57 ± 2.44 in Group I and 71.57 ± 2.29 in group II, with no statistically significant difference (p = 1.000).
  *After 6 months, implant stability increased to 80.14 ± 3.18 in group I and 81.71 ± 3.09 in group II, with no statistically significant difference between groups (p = 0.368).
  *Intra-group analysis demonstrated a statistically significant increase in implant stability from baseline to 6 months in both groups (p = 0.001) (Fig. 5).
2. Soft tissue healing
  □ At 2 weeks postoperatively, the mean Landry healing index score was 3.0 ± 0.0 in both groups, with no statistically significant difference (*p* = 1.000).
  □ At 4 weeks, the mean healing score increased to 4.38 ± 0.52 in Group I and 4.50 ± 0.53 in Group II, with no statistically significant difference (*p* = 0.642).
  □ Intra-group comparison revealed a statistically significant improvement in healing scores from 2 to 4 weeks in both groups (*p* = 0.008).

### Radiographic outcome

#### Bone volume changes

- At baseline (T0), the mean bone volume was 1065.34 ± 50.44 in Group I and 1093.74 ± 84.17 in group II, with no statistically significant difference (*p* = 0.427).
- Immediately postoperatively (T1), bone volume increased significantly in both groups, with significantly higher values observed in group II (1479.64 ± 96.27) compared to group I (1254.16 ± 35.81) (*p* = 0.001).
- At 6 months (T6), bone volume decreased compared to T1 but remained higher than baseline in both groups (1165.03 ± 39.43 in group I and 1213.34 ± 39.43 in group II), with no statistically significant difference (*p* = 0.244).
- Bone volume loss from T1 to T6 was significantly greater in group II (266.29 ± 92.52) compared to group I (92.12 ± 23.29) (*p* = 0.001).
- Intra-group analysis showed statistically significant changes over time (T0, T1, and T6) in both groups (*p* = 0.001) (Table 2).

**Table 2:** Comparison of bone volume between studied groups and during follow up

Bone volume	Group I *N* = 8	Group II *N* = 8	Test of significance
T0	1065.34 ± 50.44	1093.74 ± 84.17	t = 0.819 *P* = 0.427
T1	1254.16 ± 35.81	1479.64 ± 96.27	t = 6.13 *P* = 0.001*
T6	1165.03 ± 39.43	1213.34 ± 39.43	t = 1.22 *P* = 0.244
T1-T6( bone loss)	92.12 ± 23.29	266.29 ± 92.52	t = 5.16 *p* = 0.001*
Repeated Measures ANOVA test	F = 6263.76 *P* = 0.001*	F = 1362.35 *P* = 0.001*	
Paired comparison	P1 = 0.001* P2 = 0.001* P3 = 0.001*	P1 = 0.001* P2 = 0.001* P3 = 0.001*	

Data expressed as mean ± SD, *F* repeated Measures ANOVA test, *t* student t test

*P1* difference between T0 versus T1, *P2* difference between T0 versus T6, *p3* difference between T1 versus T6

## Discussion

The present randomized clinical trial evaluated the clinical and radiographic outcomes of Medpark bone graft and ESB for horizontal ridge augmentation with simultaneous implant placement in the anterior maxilla. The findings demonstrated that both approaches resulted in favorable implant stability, satisfactory soft tissue healing, and significant bone volume gain, with no significant differences between groups at 6 months.

Implant stability is a key determinant of osseointegration and long-term success. In the present study, a significant increase in implant stability was observed over time in both groups, indicating successful biological integration. The absence of intergroup differences suggests that horizontal ridge augmentation, regardless of graft type, does not negatively affect implant stability.

These findings are consistent with *Vollmer et al.* [29] reported a progressive increase in ISQ from placement to loading in the maxilla, reflecting successful Osseo integration. Similarly, *Al-Khaldi et al.* [30] demonstrated favorable stability in grafted anterior maxilla, with ISQ values comparable to those in native bone. *Baftijari et al.* [31] observed an increase in ISQ from 58 to 65 within three months, while *López et al.* [32]., reported an initial decrease at four weeks followed by recovery and higher values thereafter. Collectively, these results support that ridge augmentation does not compromise implant stability and allows predictable mechanical and biological integration.

Peri-implant soft tissues are essential for the long-term success of dental implants, serving as a protective barrier against microbial infiltration, enhancing aesthetic outcomes, and supporting patient comfort [33]. Both groups in this study exhibited favorable healing outcomes, with significant improvement over time and no reported complications such as dehiscence or graft exposure. The comparable healing outcomes may be attributed to the stability of the graft materials and adequate flap management. ESB, due to its fibrin-rich matrix and growth factor release [34], has been suggested to enhance soft tissue healing; however, this advantage did not translate into statistically significant clinical differences in the present study. Also, the self-hardening characteristics of Medpark bone graft form a stable foundation beneath the soft tissue, decreasing soft tissue dehiscence, hence accelerating healing. Previous studies [35, 36] have shown that synthetic bone substitutes can act as effective barriers against soft tissue ingrowth while maintaining biocompatibility and osteoconductivity. *Passi et al.* [35] reported that such materials support stable soft tissue healing without the need for additional membrane coverage, even in peri-implant dehiscence defects. *Elgendi et al.* [36]. reported progressive improvement in peri-implant soft tissue parameters from the immediate postoperative period through follow-up, with no significant differences between groups. This aligns with our findings, where healing scores increased steadily up to the fourth week, indicating effective soft tissue recovery with both sticky bone and synthetic graft approaches.

Extended Sticky Bone (Bio-Bone) promotes healing through the incorporation of PRF, whose fibrin matrix with platelets and leukocytes enables sustained release of growth factors that enhance angiogenesis, osteoblast activity, and soft tissue repair. In addition, its cohesive structure formed by the combination of PRF, Alb-gel, and particulate graft provides inherent stability and functions as a biologically active membrane, thereby eliminating the need for an additional barrier membrane during ridge augmentation [37]. 

The present results are consistent with *Alruwaili et al.* [21]., and *Aloshaysh et al.* [22] who reported optimal soft tissue healing with complete mucosal maturation and absence of complications. Similarly, no signs of infection, inflammation, or membrane exposure were observed in the current study.

Volumetric analysis offers a comprehensive evaluation of graft performance by quantifying the total three-dimensional changes in augmented bone over time. Unlike linear measurements, volumetric data reflects the biological stability and resorption dynamics of the graft material, thus offering a more accurate prediction of long-term implant support [38]. 

The present study demonstrated a significant increase in bone volume in both groups, reflecting active bone formation. Although the ESB showed greater bone volume at the early follow-up period, the difference between the two groups was not statistically significant after six months. This finding suggests that both approaches were effective in maintaining ridge volume over time.

Medpark, a moldable bovine bone mineral, exhibited a significant increase in alveolar bone volume during early healing, supporting its efficacy in ridge preservation. This may be attributed to its osteoconductive properties, while its self-hardening characteristic enhances graft stability, maintains space, minimizes micro movement [39]. The increase in bone volume in the present study is consistent with *Wang et al.* [40]., who demonstrated that xenogeneic bone grafts provide an osteoconductive scaffold that supports space maintenance and new bone formation during ridge augmentation, resulting in favorable volumetric outcomes despite ongoing remodeling.

ESB integrates Alb-gel, particulate bone graft, and injectable PRF to form a biologically active matrix with prolonged viability of 4–6 months. This sustained activity allows ESB to facilitate bone regeneration procedures without requiring additional collagen membranes, potentially reducing treatment costs. Moreover, its cohesive moldable consistency and favorable handling characteristics permit better adaptation to irregular bone defects and allow for flap tension reduction [14, 41–43]. 

Consistent with our results, *Alruwaili et al.* [21] and *Aloshaysh et al.* [22]. reported a significant increase in ridge width following horizontal bone augmentation using extended sticky bone. They observed that most of the dimensional gain occurred immediately after graft placement, with volumetric changes during the follow-up period, indicating good volume stability and effective space maintenance of the graft material during healing. In a clinical trial investigating the use of sticky bone in horizontal ridge defects, Moussa et al. [44], observed a significant increase in volume at three months, which remained stable at six months. This effect was attributed to the fibrin matrix within the sticky bone, which serves as a scaffold that slows resorption and facilitates mineralized bone formation. Likewise, the addition of concentrated growth factors to sticky bone has been reported to decrease volumetric resorption and enhance bone maturation [44]. 

From a clinical view, the choice between ESB and Medpark bone graft may depend on specific treatment priorities. ESB may offer advantages in handling, adaptability, and early graft stabilization, particularly in irregular defects. On the other hand, Medpark may be preferred in cases where long-term volumetric stability is critical. Therefore, both materials represent viable options, and their selection should be tailored to individual clinical scenarios.

In the present study, equal amounts of particulate bone grafts were used in both groups. However, in ESB group, the xenograft particles were mixed with ALB-PRF to form a biologically active matrix with increased overall volume. Subsequently, significantly higher volumetric measurements were observed immediately after surgery, likely reflecting not only the particulate xenograft but also the additional ALB-PRF incorporated within ESB.

During the healing period, the ESB group revealed greater volumetric bone reduction than the Medpark group. This finding may be explained by the gradual degradation of the fibrin matrix, which is designed to act as a temporarily biologically active matrix that promotes tissue regeneration. In contrast, Medpark is a self-hardening bovine-derived graft characterized by greater structural rigidity and resistance to dimensional changes, which may have contributed to the superior volumetric stability observed during the healing period. Furthermore, the larger initial augmented bone volume achieved with ESB may have resulted in increased tension within the overlying soft tissues during the healing period, which may contribute to the higher volumetric reduction observed between T1 and T6. Similar results were reported in previous studies evaluating ESB, where greater early bone graft volume was followed by volumetric changes during the healing period [21, 22]. 

This resorption pattern suggests that early volumetric expansion alone may not be an appropriate indicator of long-term clinical performance and highlights the importance of evaluating both initial augmentation capacity and dimensional stability over time.

Future studies with larger sample sizes, longer follow-up periods, and histological analyses are warranted to evaluate the long-term dimensional stability of the augmented ridge, implant survival, and treatment outcomes, and to determine whether the early differences observed between the grafting approaches are associated with clinically meaningful benefits over time.

## Conclusion

Within the limitations of this study, both Medpark and extended sticky bone demonstrated favorable short-term outcomes for horizontal ridge augmentation. Although extended sticky bone achieved greater immediate postoperative bone volume gain, greater subsequent resorption resulted in comparable clinical and radiographic outcomes between the two groups after 6 months. Further studies with larger sample sizes and longer follow-up periods are required to determine the long-term clinical significance of these findings.

## Data Availability

The data sets used and/or analyzed during the current study are available from the corresponding author on reasonable request.
